# 16S rDNA analysis of periodontal plaque in chronic obstructive pulmonary disease and periodontitis patients

**DOI:** 10.1080/20002297.2017.1324725

**Published:** 2017-06-12

**Authors:** Xingwen Wu, Jiazhen Chen, Meng Xu, Danting Zhu, Xuyang Wang, Yulin Chen, Jing Wu, Chenghao Cui, Wenhong Zhang, Liying Yu

**Affiliations:** ^a^ Department of Dentistry, Huashan Hospital, Fudan University, Shanghai, P.R. China; ^b^ Department of Dentistry, Zhongshan Hospital, Fudan University, Shanghai, P.R. China; ^c^ Department of Infectious Diseases, Huashan Hospital, Fudan University, Shanghai, P.R. China

**Keywords:** Oral microbiota, subgingival plaque, high-throughput sequencing, 16s rRNA gene, chronic obstructive pulmonary disease, chronic periotontal disease

## Abstract

This study investigated if chronic obstructive pulmonary disease (COPD) is correlated with periodontitis via periodontal microbiota and if certain bacteria affect periodontitis as well as COPD. Moreover, the study investigated whether suffering from COPD is associated with a decrease in the richness and diversity of periodontal microbiota. Subgingival plaque was obtained from 105 patients. Bacterial DNA was isolated from 55 COPD and 50 non-COPD participants (either with or without periodontitis). 16S rRNA gene metagenomic sequencing was used to characterize the microbiota and to determine taxonomic classification. In the non-periodontitis patients, suffering from COPD resulted in a decrease in bacteria richness and diversity in the periodontal microenvironment. An increase in the genera *Dysgonomonas*, *Desulfobulbus*, and *Catonella* and in four species (*Porphyromonas endodontalis*, *Dysgonomonas wimpennyi*, *Catonella morbi*, and *Prevotella intermedia*) in both COPD and periodontitis patients suggests that an increase in these periodontitis-associated microbiota may be related to COPD. Three genera (*Johnsonella*, *Campylobacter*, and *Oribacterium*) were associated with COPD but not with periodontitis. The decrease in the genera *Arcanobacterium*, *Oribacterium*, and *Streptomyces* in COPD patients implies that these genera may be health-associated genera, and the decrease in these genera may be related to disease. These data support the hypothesis that COPD is correlated with periodontitis via these significantly changed specific bacteria.

## Introduction

Chronic periodontitis is a common oral disease, with symptoms ranging from gingival bleeding and clinic attachment loss to periodontal abscess and even tooth loss. Recent research has established that periodontal infection is a probable risk factor for diabetes mellitus, cardiovascular disease and atherosclerosis, stroke, adverse pregnancy outcomes, and respiratory disorders including chronic obstructive pulmonary disease (COPD) [1].

COPD can be characterized by progressive deterioration of pulmonary function and increasing airway obstruction, including chronic bronchitis and emphysema. Accumulating evidence suggests that oral disorders, particularly periodontal disease, may influence the course of respiratory infections such as bacterial pneumonia and COPD [2,3]. Periodontitis is positively associated with COPD [4], and periodontal probe depth is identified as a significant and independent risk factor for COPD [5]. Treating periodontitis in COPD patients resulted in higher measurements of lung function and lower frequencies of COPD exacerbation up to 2 years after receiving standard periodontal treatment [6].

Lung tissue is not a sterile environment [7], but the sources of lung microorganisms are still being identified. The bacteria of the lung reflect ‘immigration’ [8] via inhalation of air, direct mucosal dispersion, and microaspiration [9–11]. This is especially true in healthy lungs through microaspiration from a healthy oral microenvironment, which suggests a great association of microbiota in oral and respiratory tissues in healthy individuals [12,13]. However, the association of microbiota in COPD patients remains unclear. The periodontal pocket provides a suitable microenvironment for both pathogenic and opportunistic species of bacteria, and this increases the risk of aspirating pathogenic bacteria into the lung and causing pneumonia [14]. The local predisposing factors of chronic periodontitis can be present in the periodontal pocket, mainly the subgingival non-adherent plaque. The statistical association between periodontitis and COPD has been clinically established [5,6], but less is known regarding how they are associated and their underlying mechanisms. In a previous study, some specific bacteria including *Klebsiella pneumonia*, *Pseudomonas aeruginosa*, *Streptococcus pneumoniae*, *Porphyromonas gingivalis*, *Treponema denticola*, and *Tannerella forsythia* were more abundant in both tracheal aspirate and periodontal pockets in severe acute exacerbations of COPD patients, suggesting particular bacteria were related to acute exacerbations of COPD [15].

This study hypothesized that COPD is correlated with periodontitis via periodontal microbiota and that certain specific bacteria affect periodontitis as well as COPD. Moreover, it proposed that suffering from COPD is associated with a decrease in the richness and diversity of periodontal microbiota. The richness, diversities, and relative abundance of periodontal bacteria were compared among COPD patients, periodontitis patients, and a control population by using a 16S rRNA gene metagenomic sequencing technique that has been widely used to characterize human microbiota in various tissues [16]. The study also investigated whether some specific bacteria were associated with COPD and periodontitis simultaneously by overlapping the significantly changed bacteria in COPD with those in periodontitis.

## Methods

### Patient recruitment

This study was observational and paralleled in design. A total of 105 participants (average age 64.8 ± 7.0 years) were recruited from March 2014 to June 2015 and formed four groups: 30 patients with periodontitis and COPD (C+P+ group), 25 COPD patients without periodontitis (C+P− group), 25 periodontitis patients without COPD (C−P+ group), and 25 healthy individuals (C−P− group). Because most patients with COPD are older adults and age could be a factor in affecting periodontal bacteria composition [17], the non-COPD groups were age-matched to the COPD groups. This study conforms to STROBE Guidelines. All subjects provided informed consent before enrollment in the study. Ethics approval was obtained from the Ethics Committee of Huashan Hospital, Fudan University (no. KY2014-023). The study design is shown in Appendix Figure 1.

**Figure 1.**
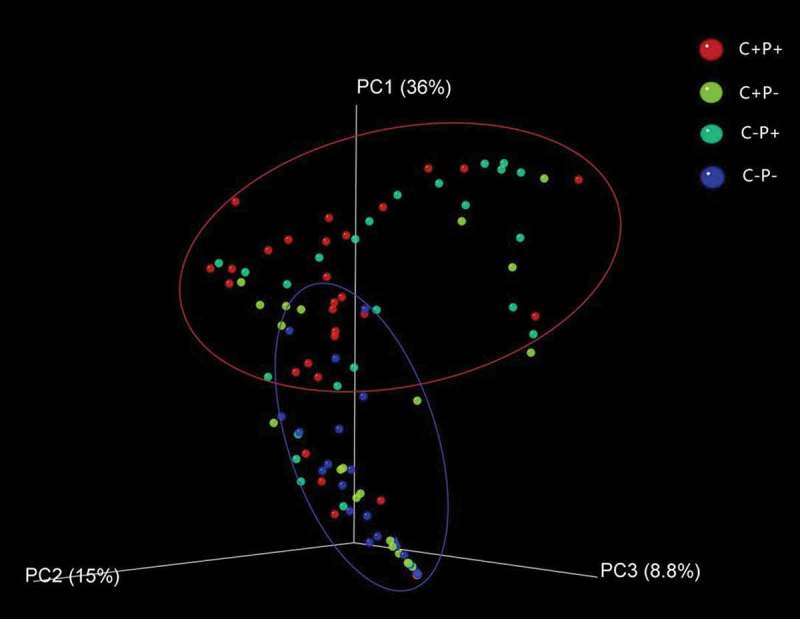
Beta diversity analysis based on weighted UniFrac analysis. Red dots represent periodontitis patients with chronic obstructive pulmonary disorder (COPD; C+P+ group), green dots represent periodontitis patients without COPD (C−P+ group), yellow dots represent COPD patients without periodontitis (C+P− group), and blue dots represent participants without COPD or periodontitis (C−P− group). Red and blue circles represent different periodontal bacterial community clusters.

COPD patients were initially identified by a review of medical histories, and spirometry examination subsequently confirmed the diagnosis of COPD. COPD status was estimated according to previously published criteria [18]. Only stable moderate and severe COPD patients without systemic disease (such as diabetes and atherosclerosis) were recruited. Patients who had consumption of systemic antimicrobials in the prior month or acute exacerbations in the previous 3 months were excluded. Probing depth (PD), clinical attachment loss (CAL), and simplified oral hygiene index (OHI-S) were assessed according to World Health Organization recommendations [19]. Periodontitis was defined as the presence of ≥1 tooth with at least one site with PD ≥4 mm, CAL ≥2 mm, and bleeding on probing. The diagnosis was made for at least 30% of all sites demonstrating these symptoms. Patients with periodontitis ranged from moderate (PD = 4–5 mm; CAL = 3–4 mm) to severe (PD >5 mm; CAL ≥5 mm) were recruited. Patients who had mild periodontitis (PD = 3–4 mm; CAL = 1–2 mm) or periodontal therapy in the previous 3 months were excluded.

### Sample collection and isolation of bacterial DNA

After scratching the supragingival debris with sterilized cotton balls, subgingival plaque was scraped with pre-sterilized No. 25 paper points (Gapadent, China) and stored in Tris-EDTA buffer, pH 7.4 (Sigma–Aldrich, St. Louis, MO). The mesiobuccal pocket of the maxillary first molar was sampled. If the maxillary first molar was missing, the maxillary second molar or mandibular molars were sampled instead. In individuals without periodontitis, periodontal plaque was extracted from the mesiobuccal gingival crevice of a periodontally healthy molar. Bacterial DNA was extracted using the QiAamp DNA Mini Kit (Qiagen, Hilden, Germany) following the manufacturer’s instructions.

### Amplification of the 16S rDNA by polymerase chain reaction

The V4–V5 regions [20] of 16S rDNA were amplified from bacterial DNA using a forward primer (515F: GTG CCA GCM GCC GCG GTA A); and a reverse primer (927R: KCC CCC GTC AAT TCC TTT RAG TTT) [21]. The amplification, polymerase chain reaction (PCR) product clean-up and index PCR followed the protocol ‘16S Metagenomic Sequencing Library Preparation’ (http://support.illumina.com/downloads/16s_metagenomic_sequencing_library_preparation.html), except that the PCR clean-up was substituted by using the MiniElute PCR Purification Kit (Qiagen) and the MiniElute Gel Extraction Kit (Qiagen). The dsDNA concentration was measured by a Qubit 2.0 fluorometer (Invitrogen, Carlsbad, CA).

### Sequencing 16S rDNA

Equal amounts of the tagged 16S rRNA gene amplicons were mixed and denatured with 0.1 M of NaOH. The mixed library was diluted to a final concentration of 10–20 pM using 10 mM of Tris, pH 8.5. Multiplexed paired-end sequencing (2 × 300 bp reads) of the 16S rRNA amplicons was performed using a Miseq system (Illumina, San Diego, CA).

Image analysis and base calling were done on the Miseq system using the MiSeq Reporter software (MSR). After de-multiplexing the data and removal of reads that failed Illumina’s purity filter (PF  =  0), reads were converted to FASTQ format.

### Data analysis and statistics

The Illumina-generated FASTQ files (.fastq) and quality files were acquired as raw and mapped sequence data. Each operational taxonomic unit (OTU) was generated using the default settings in the QIIME software (v1.9.1) [22]. Classifications were based on the Greengenes database (http://greengenes.lbl.gov/) [23]. The output classified the reads at several taxonomic levels. Alpha- and beta-diversity analyses were computed from the previously constructed OTU table using Mothur software (v1.21.1) and weighted UniFrac analysis. Abundance analysis was determined from rarefaction files using the Kruskal–Wallis test among four groups and the Mann–Whitney test between each of two groups (IBM SPSS Statistics for Windows v20 and GraphPad Prism software, v6.01). Additionally, weighted Student’s *t*-tests were performed between the COPD (C+P+ and C+P− groups) and non-COPD (C−P+ and C−P− groups) patients. The minimum abundance cut-off was set at 0.1% abundance.

## Results

### Demographic data

The 105 study participants were divided into four groups, and the demographic data for each group are described in Table 1. Except for sex, smoking, and OHI-S, no other differences were observed in terms of demographics. In this study, non-smokers either never smoked or quit cigarettes at least 10 years prior to study entry [24]. There was a difference in smokers and non-smokers among the four groups (χ^2^ = 8.291; *p* < 0.05), and the smoker percentage in the C+P+ group was higher than that in the C+P− group. However, there were no statistical differences in smokers and non-smokers between the C+P+ and C+P− groups (χ^2^ = 1.516; *p* > 0.05), C+P+ and C−P+ groups (χ^2^ = 3.346; *p* > 0.05), C+P− and C−P− groups (χ^2^ = 2.381; *p* > 0.05), and C−P+ and C−P− groups (χ^2^ = 0.936; *p* > 0.05). The scores of OHI-S in periodontitis patients were statistically higher in non-periodontitis participants. Although the severity percentage in the C+P+ group was higher than that in the C+P− group, there was no statistically significant difference between those two groups (χ^2^ = 2.013; *p* > 0.05), and the FEV1 value (represented the severity of the disease) between the two groups was also not different (*p* > 0.05).

**Table 1.** Characteristics and taxonomic data of enrolled study participants.

	C+P+	C+P–	C–P+	C–P–
Sex (%)				
Male	24 (80.0%)	17 (68.0%)	19 (76.0%)	11 (44.0%)
Female	6 (20.0%)	8 (32.0%)	6 (24.0%)	14 (56.0%)
Age				
Mean (*SD*)	65.2 (7.4)	65.6 (7.1)	63.4 (7.0)	64.8 (6.7)
Smoking status (%)				
Non-smoker^a^	13 (43.3%)	15 (60.0%)	17 (68.0%)	20 (80.0%)
Smoker	17 (56.7%)	10 (40.0%)	8 (32.0%)	5 (20.0%)
Former smoker^b^	4 (13.3%)	5 (20.0%)	1 (4.0%)	1 (4.0%)
Current smoker^c^	13 (43.3%)	5 (20.0%)	7 (28.0%)	4 (16.0%)
Cigarettes/day (mean ± *SD*)				
Non-smoker	0 ± 0	0 ± 0	0 ± 0	0 ± 0
Smoker	19.4 ± 12.1	22 ± 7.9	20.5 ± 13.6	16.4 ± 14.4
Former smoker	13.8 ± 7.5	24 ± 11.4	20 ± N/A	20 ± N/A
Current smoker	21.8 ± 12.8	20 ± 0	20.6 ± 14.6	15.5 ± 16.5
COPD classification (%)^d^				
II	16 (53.3%)	18 (72.0%)	/	/
III	14 (46.7%)	7 (28.0%)	/	/
FEV1				
Mean (*SD*)	51.9 (14.1)	59.3 (14.3)	/	/
Inhaled steroids	4 (13.3%)	4 (16.0%)	/	/
OHI-S				
Mean (*SD*)	2.21 (0.32)	1.52 (0.42)	2.18 (0.43)	1.45 (0.51)
Clinical attachment loss (mm)				
Mean (*SD*)	5.5 (0.7)	/	5.9 (1.2)	/
Detected sequence (thousands)				
Mean (*SD*)	132 (83)	204 (63)	178 (64)	175 (83)
Classified sequence (%)				
Mean (*SD*)	94.83 (0.35)	94.61 (1.26)	94.8 (0.59)	94.75 (0.46)

^a^Non-smokers were those who either had never smoked or quit cigarettes at least 10 years prior to study entry.

^b^Former smokers were those who quit cigarettes at least 6 months but <10 years prior to study entry.

^c^Current smokers were currently smokers or those who quit cigarettes <6 months prior to study entry.

^d^Patients with COPD were grouped into moderate (II predicted FEV1 = 50–80%; FEV1/FVC ≤70%) and severe (III, predicted FEV1 = 30–50%; FEV1/FVC ≤70%) categories based on spirometry.

### Taxonomic classification of 16S rDNA sequences

Approximately 16.9 million high-quality and classifiable reads were obtained from the oral microflora isolated from 105 participants (Table 1). Among these high-quality reads, 94.63% were taxonomically classified into 198 genera belonging to 17 phyla, 29 classes, 56 orders, and 109 families. Among the four groups, no obvious bias was observed in the proportion of unclassifiable sequences (*p* = 0.715).

## Microbial communities in the periodontal pocket and gingival crevice

### Alpha-diversity analysis

Plots were generated and exported for the rarefaction curves [25] (subsampled, Appendix Figure 2). Sequencing data from all four groups produced saturatedly approximately at 80,000 reads, and no differences were observed (Appendix Figure 2). Based on the Shannon index, representing diversity, and Chao1 index, representing richness, of the four groups (Appendix Figure 3), the data showed a significantly decreased richness and diversity in the C+P− group compared to the C−P− group, but no significant difference was found between the C+P+ and C−P+ groups. The data suggested that in non-periodontitis patients, suffering from COPD resulted in decreasing bacteria richness and diversity in the periodontal microenvironment.

**Figure 2.**
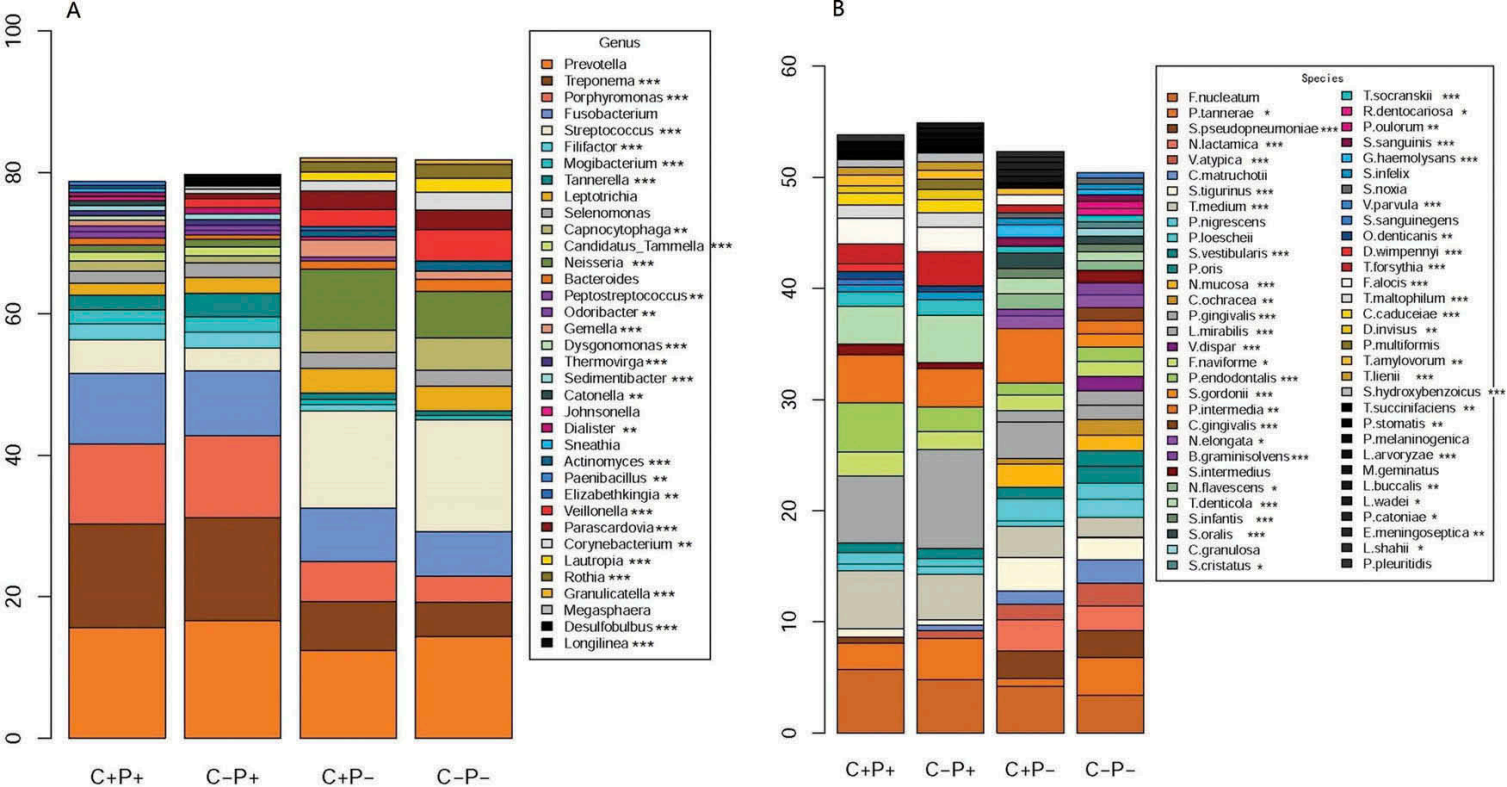
Relative abundance and composition of microbial genera (**A**) and species (**B**) in four groups of participants. The abundance cut-off in this figure was set at 0.5%. Some values <0.5% were not shown. A Kruskal–Wallis test was used to analyse diversity among the four groups. Statistical significance is indicated by the following: *p < 0.05; **p < 0.01; ***p < 0.001.

**Figure 3.**
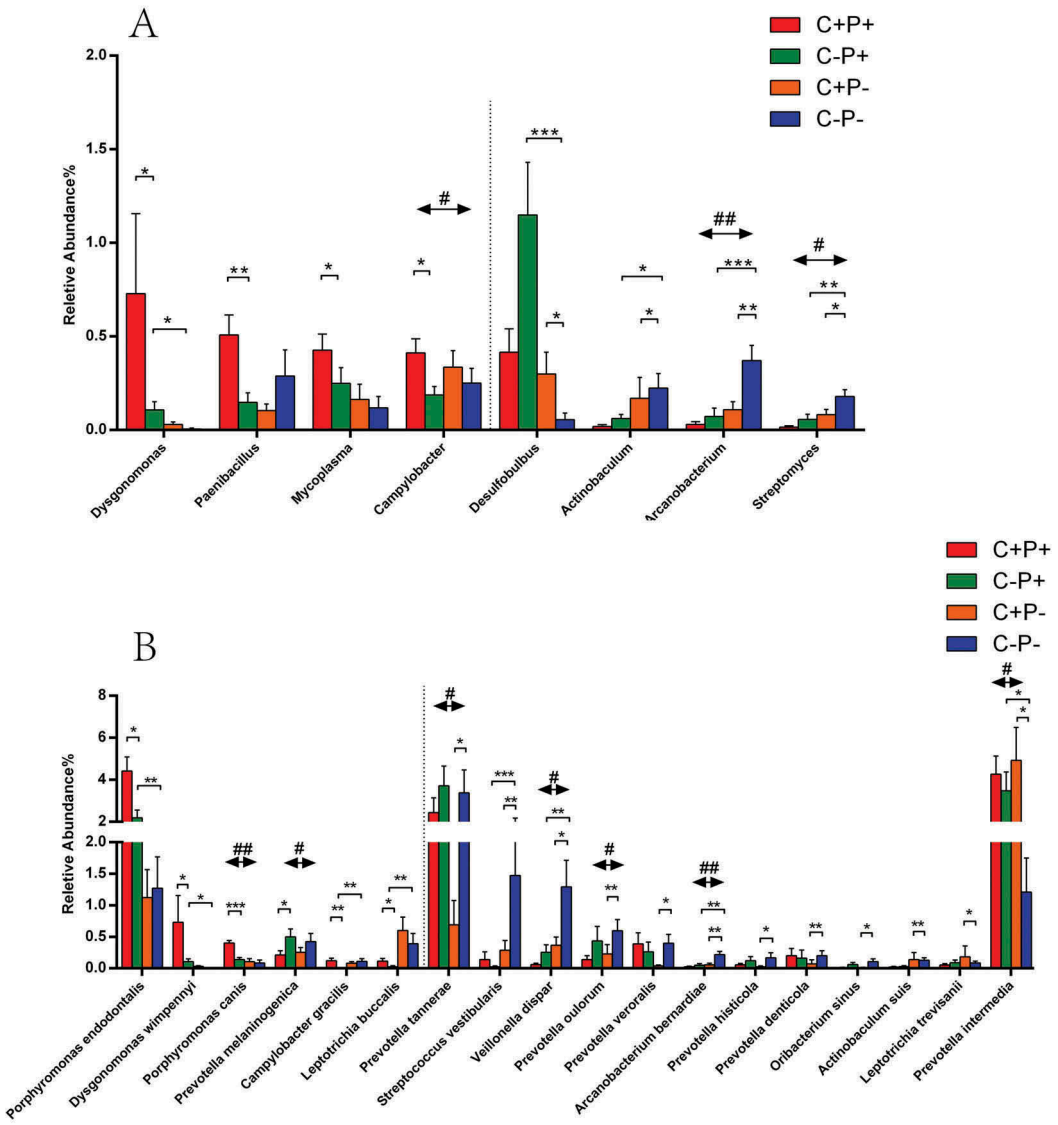
Mean relative abundance of bacterial genera (**A**) and species (**B**) with statistical differences between COPD and non-COPD patients. Data from COPD patients (C+P+ and C+P−) and non-COPD patients (C−P+ and C−P−) are shown. The abundance cut-off was set at 0.1%. Some values <0.1% were not calculated or shown in the figure. Statistical significance is indicated by the following: #*p* < 0.05, significant difference between COPD and non-COPD patients using weighted Student’s *t*-tests; ##*p* < 0.01; ###*p* < 0.001; *p < 0.05; **p < 0.01; ***p < 0.001.

### Beta-diversity analysis

The microbial raw OTU dates were subjected to principal coordinates analysis (PCoA) to evaluate the similarities among the four groups. Periodontal pocket samples from the C+P+ and C−P+ groups had similar microbial compositions, which can be grouped together into one cluster, and gingival crevice samples from the C−P− group made another cluster (Figure 1). The samples from the C+P− group could not be grouped into one cluster but was scattered in the 3D plot (Figure 1). The data suggest that the periodontitis patients had similar bacterial compositions in periodontal tissue irrespecitve of whether they were suffering from COPD. However, interestingly, the bacterial composition of COPD patients without periodontitis was scattered and did not differ from either periodontitis-afflicted or healthy individuals.

### Abundance analysis

A total of 198 bacterial genera were detected in the 105 oral periodontal samples. On average, the abundance of 69 genera and 111 species was >0.1%. Those abundant genera and species are listed in Appendix Tables 1, 2, and 2B.

In agreement with the beta-diversity analysis, where the C+P+ and C−P+ group had similar microbial compositions, the dominant species were similar in the two groups. *P*. *gingivalis*, followed by *Fusobacterium nucleatum*, *Treponema medium*, and *T*. *denticola*, were the most dominant species in these two groups (Figure 2).

In the C+P− and C−P− groups, the dominant bacterial species were different. In the C+P− group, the most abundant species were *Prevotella intermedia*, *F. nucleatum*, *P. gingivalis*, and *Streptococcus tigurinus*, while in the C−P− group, the most abundant species were *F*. *nucleatum*, *Prevotella tannerae*, *Streptococcus pseudopneumoniae*, and *Neisseria lactamica* (Figure 2).

### Specific bacteria associated with periodontitis in subgingival plaque

**Figure 4.**
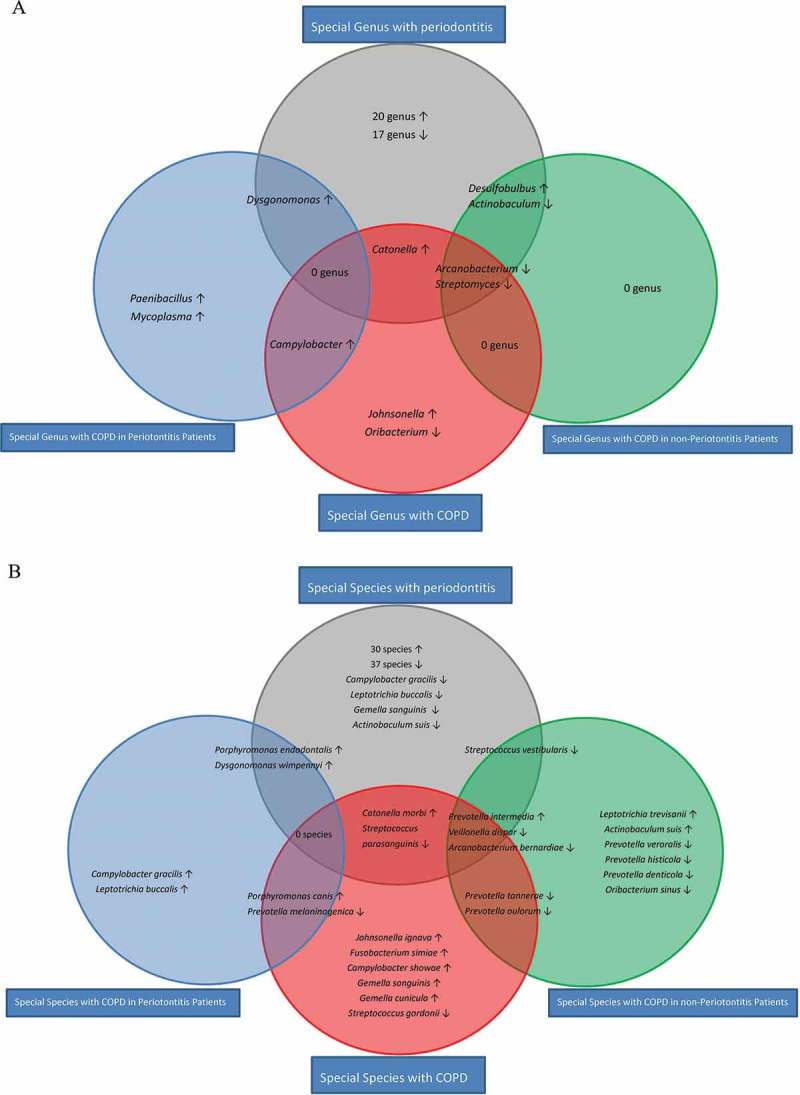
Statistically significant bacterial genera (**A**) and species (**B**) associated with COPD and periodontitis in periodontitis and non-periodontitis patients. The red circle represents significantly different bacterial abundance between COPD and non-COPD patients. The blue circle represents significantly different bacterial abundance between C+P+ and C−P+ groups. The green circle represents significantly different bacterial abundance between C+P− and C−P− groups. The gray circle represents significantly different bacterial abundance between C−P+ and C−P− groups. No overlapping area between blue and green circles was observed. The abundance cut-off was set at 0.1%. Some values <0.1% were not calculated or shown in the figure. ↑, increased; ↓, decreased.

A comparison between the microbial abundance in the C−P+ and C−P− groups was made. The abundance of 23 genera and 34 species was higher in the C−P+ group than it was in the C−P− group. The abundance of 20 genera and 45 species was lower in the C−P+ than it was in the C−P− group. (Figure 4, Appendix Tables 4 and 5). These increased and decreased bacteria may be associated with chronic periodontitis.

### Specific bacteria associated with COPD in subgingival plaque

A comparison was made of the microbial abundance between COPD and non-COPD patients. The abundance of six genera and 15 species was significantly different, suggesting these bacteria could be associated with COPD. Among them, the genera *Johnsonella*, *Catonella*, and *Campylobacter*, as well as the species including *P*. *intermedia*, *Porphyromonas canis*, *Johnsonella ignava*, and *Catonella morbi*, were more abundant in COPD than they were in non-COPD participants. Contrarily, the abundance of the genera *Arcanobacterium*, *Oribacterium*, and *Streptomyces*, as well as species including *P*. *tannerae*, *Veillonella dispar*, *Arcanobacterium bernardiae*, and *Prevotella melaninogenica*, were lower in COPD than in non-COPD participants (Figures 3 and 4 and Appendix Table 3).

When comparisons were made between C+P+ and C−P+ groups as well as C+P− and C−P− groups, the results of the analyses were distinct between periodontitis population and non-periodontitis population, with no overlapping bacteria observed (Figure 4). Therefore, the abundant differences in the two subpopulations were analyzed separately: the periodontitis population and the non-periodontitis population. In periodontitis patients, four genera (*Dysgonomonas*, *Paenibacillus*, *Mycoplasma*, and *Campylobacter*) and five species (*Porphyromonas endodontalis, Dysgonomonas wimpennyi, P. canis, Campylobacter gracilis*, and *Leptotrichia buccalis*) were higher, and only one species (*P*. *melaninogenica*) was lower in the C+P+ group compared to the C−P+ group (Figures 3 and 4). However, in non-periodontitis participants, the abundance of only one genus (*Desulfobulbus*) and three species (*P*. *intermedia*, *Leptotrichia trevisanii*, and *Actinobaculum suis*) were higher in C+P− patients than in C−P− healthy controls. The three genera (*Actinobaculum*, *Arcanobacterium*, and *Streptomyces*) and nine species (*P*. *tannerae*, *Streptococcus vestibularis*, *V. dispar*, *Prevotella oulorum*, *Prevotella veroralis*, *A. bernardiae*, *Prevotella histicola*, *Prevotella denticola*, *and Oribacterium sinus*) were lower (Figures 3 and 4).

### Bacteria associated with both COPD and periodontitis

Among these bacteria associated with periodontitis, three genera and five species were different in COPD patients (Figure 4; the overlapping area in red and gray). The genus *Catonella* and two species (*C*. *morbi* and *P*. *intermedia*) predominated in COPD patients compared to non-COPD patients. Contrarily, two genera (*Arcanobacterium* and *Streptomyces*) and three species (*Streptococcus parasanguinis*, *V. dispar*, and *A*. *bernardiae*) were less abundant.

Similarly, different data were shown between the COPD patients with and without periodontitis. Among the bacteria associated with periodontitis, four genera and four species were different in non-periodontitis COPD patients (Figure 4; the overlapping area in green and gray). Only one genus (*Desulfobulbus*) and one species (*P*. *intermedia*) were higher in the C+P− group than in the C−P− group, while three genera (*Actinobaculum*, *Arcanobacterium*, and *Streptomyces*) and three species (*S*. *vestibularis*, *V. dispar*, and *A. bernardiae*) were lower.

Additionally, among the bacteria associated with periodontitis, the abundance of only one genus (*Dysgonomonas*) and two species (*P*. *endodontalis* and *D*. *wimpennyi*) were higher in the C+P+ group than in the C−P+ group. No decrease in bacteria was observed (Figure 4; the overlapping area in blue and gray).

The increasing bacteria in both COPD and periodontitis suggests their possible roles in both diseases, and the decreasing bacteria suggests their possible role in maintaining healthy status.

Interestingly, three genera (*Johnsonella*, *Campylobacter*, and *Oribacterium*) and five species (*J*. *ignava*, *P. canis*, *Fusobacterium simiae*, *Campylobacter showae*, and *Gemella cunicula*) were found to be associated with COPD but not with periodontitis (Figure 4; red circle).

## Discussion

Periodontal microbiota composition has been widely analyzed in recent years [26]. In the present study, *P*. *gingivalis*, *F. nucleatum*, *T. denticola*, *T. medium*, and *P. intermedia* were more abundant in periodontal patients compared to healthy participants. This result was similar to observations from previous studies [27], indicating the dominant microbiota in periodontal patients was relatively stable in diverse populations, and strengthened the reliability of the data sampling periodontal tissue in COPD patients.

In this study, six periodontitis-associated genera and eight species were identified in COPD patients. The genera *Dysgonomonas*, *Desulfobulbus*, and *Catonella*, as well as *P*. *intermedia*, *P. endodontalis*, *D. wimpennyi*, and *C*. morbi, were more abundant in COPD patients than they were in non-COPD patients. The increasing abundance of these microorganisms in periodontal tissue could be related to the development of COPD.

The genera *Prevotella* and especially *P*. *intermedia* [28] are the most dominate anaerobes isolated from the periodontal abscess. In this study, *P*. *intermedia* was more abundant in COPD patients than it was in non-COPD participants, suggesting that the bacteria could also be associated with COPD. *In vivo*, *P. intermedia* invades the gingival epithelial cells [29] and induces strong expression of antimicrobial peptides and interleukin-8 [30]. These events lead to the migration of innate immune cells to the local infections and bactericidal activity, resulting in an imbalance in the local flora and severe inflammation [31]. COPD develops as a significant and chronic inflammatory response to inhaled irritants [32], in which bacteria can contribute to the inflammatory state [33]. As pathogens or opportunistic pathogens from the oral cavity can be inhaled, chronic bacterial infection in the oral space may add to the pulmonary inflammation.

*P*. endodontalis is a pathogenic microorganism in oral tooth perioapical infections. This species was identified as being more abundant in the C+P+ group than in the C−P+ group. A recent study suggests that *P*. *endodontalis* is responsible for periodontal inflammation and bone reabsorption [34], which is corroborated by the present data that shows that the species may be related to both COPD and periodontitis in the oral periodontal microenvironment.

The genus *Dysgonomonas* is a gram-negative anaerobe which can be isolated from the human intestine [35], brain abscesses [36], and bronchoalveolar lavage fluid (BALF) from COPD patients [14]. Few reports have characterized *D*. *wimpennyi*. Recent studies have implicated the genus *Desulfobulbus* as a cause of periodontitis [37,38] and an increased genus in BALF from COPD patients [14], which is also supported by the present results. Simultaneously, *Catonella* may contribute to pulmonary disease, such as tuberculosis [23] and cystic fibrosis [39]. In this study, the genus *Catonella* and *Catonella mordi* were more abundant in COPD patients and higher in periodontitis patients. The three genera may play an important role in respiratory cavity infections in both oral and pulmonary cavities.

This study demonstrated that the genera *Johnsonella* and *J*. *ignava* could be uniquely associated with COPD but not with periodontitis. A study recently suggested that *Johnsonella* and *J*. *ignava* were associated with oral squamous-cell carcinoma [40]. The genus *Johnsonella* may be an opportunistic pathogen, but further studies are needed. In addition to pathogenic-associated periodontal microbiota, this study also revealed the healthy-associated periodontal bacteria. In COPD patients, a decrease in the genera *Oribacterium*, *Streptomyces*, and *Arcanobacterium* was detected in the gingival crevice and periodontal pockets compared to non-COPD participants, indicating that these bacteria could be normal flora in the periodontium in non-COPD participants. The genus *Oribacterium* was detected to be significantly decreasing in sputum samples from advanced COPD patients compared to moderately severe COPD patients [41], which supported the results. In BALF samples, a statistically significant decrease in the genus *Streptomyces* was found in stable COPD patients compared to healthy individuals [42], which is also corroborated by the present data obtained from sampling dental plaque. However, in another study, *A*. *bernardiae* was found to be increased in brain abscesses [43]. This research does not coincide with the results from the present study. One possible explanation for the discrepancy is that the previous study utilized antibiotics, which resulted in a decrease in some pathogens, suggesting the species may be considered an opportunistic pathogen. COPD may relate to the imbalance of normal periodontal flora, resulting in a decrease in some beneficial species that provide a protective role to the host. The imbalance within normal flora makes the host vulnerable to the attack of pathogenic bacteria, which can lead to disease.

Smoking is a risk factor for periodontitis and can affect the periodontal microbiota [24,44]. Meanwhile, smoking is also a main risk factor for COPD [14], but has weak impact on the lung microbiota in healthy individuals [10]. Recruiting COPD patients without a recent smoking history was difficult. In this study, non-smokers versus smokers did not differ statistically in four primary comparisons. Previous research showed that species including *Parvimonas micra*, *C. gracilis*, *Treponema socranskii*, *Dialister pneumosintes*, *Streptococcus sanguinis*, and *T*. *forsythia* were significantly elevated in smoking periodontitis patients than they were in non-smoking periodontitis patients [44], and species including *F*. *nucleatum* and *Filifactor alocis* were increased in smoking periodontal healthy individuals than they were in healthy non-smokers [45]. Among these species, only *C*. *gracilis* overlapped with the present results, as it was higher in the C+P+ group than it was in the C−P+ group. *F. nucleatum* and *F. alocis* were found to be associated only with periodontitis in the present results, which suggesting that smoking status did not influence the results substantially if at all.

This study has several limitations. First, fewer male individuals were recruited in the healthy control group compared to the other groups due to the difficulty in recruiting healthy male individuals without COPD or periodontitis in older patients. Second, more current/former smokers were recruited in the C+P+ group than in the C+P− group. Although non-smokers versus smokers did not statistically differ between the C+P+ and C+P− groups, the status of more current/former smokers in the C+P+ group may have also influenced the results. According to the age-matched recruiting strategy in the study, patients around 60 years of age were recruited. In that age group, most people have some form of periodontitis, and the periodontitis was highest in men and current smokers [46]. Male non-smokers with COPD or periodontitis were difficult to recruit to match the C+P+ group in this study, so a large sample size will be used in future research to minimize this bias. Third, lower airway microbiota was not sampled in this study. Although the microbiota in periodontal tissue can immigrate into lower airway tracts and some dominant pathogenic bacteria were detected both in periodontal pocket and tracheal aspirate [15], to what extent the periodontal bacteria is associated with that in lower airway is less understood. Lower airway microbiota was the most optimal and direct way to study the microbiota related to COPD. Sampling BALF and comparing its microbiota with periodontal microbiota could be the most beneficial way to study microbiota in COPD and periodontitis. However, it is difficult to sample enough BALF from non-COPD participants because of the invasive and challenging surgical procedure that is required. Therefore, BALFs were not sampled in this study. Fourth, long-term use of inhaled steroids may cause oral disease such as periodontitis and oral caries [47], with likely changes in oral microbiota. In this study, patients who consume inhaled steroids presented 13.3% of the C+P+ group and 16% of the C+P− group, which is 14.5% of the overall COPD group and may bias the results.

In this study, only bacteria in periodontal tissue were detected, and some significant pathogens were found in the periodontal pocket, which may be related to COPD. Meanwhile, some healthy-associated periodontal bacteria were found, and these may contribute to the balance of normal flora. Compared to previous research [15], two differences were evident. First, this study concentrated on stable COPD, which was widely distributed in older patients. In addition, it focused on the periodontal bacteria that affected both COPD and periodontitis and not the bacterial relationships between dental plaques and tracheal aspirate in acute exacerbations of COPD patients. Therefore, stable COPD patients and healthy controls (with or without periodontitis) were recruited to make comparisons of COPD and healthy controls as well as periodontitis and healthy controls to identify both COPD- and periodontitis-associated bacteria in periodontal tissue. This study presents a comprehensive assessment of the effects of stable COPD on subgingival bacterial and may allow surveillance of COPD via detection of periodontal tissue.

## Summary

In summary, a total of 198 bacterial genera were detected within 105 participants, and the abundance of 69 genera and 111 species was >0.1%. Suffering COPD decreases the bacterial richness and diversity in periodontal tissue in individuals without peridontitis. The increase in the genera *Dysgonomonas*, *Desulfobulbus*, and *Catonella*, as well as *P*. *endodontalis*, *D. wimpennyi*, *C. morbi*, and *P*. *intermedia*, are associated with both COPD and chronic periodontitis. The increase in the genus *Johnsonella* may be associated with COPD and not with periodontitis. A decrease in the genera *Oribacterium*, *Streptomyces*, and *Arcanobacterium* was observed in the periodontal tissue of COPD patients. Recognition of the microbial association and differences between COPD and periodontitis may reveal new strategies for the diagnosis, surveillance, and treatment of COPD, including the accurate use of antibiotics and probiotics.

## Supplementary Material

revised-Suppl_Appendix_FIN.docx
