# Supplementary material for: 16S rDNA analysis of periodontal plaque in chronic obstructive pulmonary disease and periodontitis patients
Source: J Oral Microbiol. 2017 Jun 12;9(1):1324725. doi: 10.1080/20002297.2017.1324725 (PMC5508401; doi:10.1080/20002297.2017.1324725)
Supplement: revised-Suppl_Appendix_FIN.docx [file zjom_a_1324725_sm1407.docx]

Supplemental Appendix

**16S rDNA analysis of periodontal plaque in COPD and periodontitis patients**

Xingwen Wu^1, 3^, Jiazhen Chen^2,^*, Meng Xu^1^, Danting Zhu^1^, Xuyang Wang^2^, Yulin Chen^2^, Jing Wu^2^, Chenghao Cui^1^, Wenhong Zhang^2^, Liying Yu^1,^*

^1^Department of Dentistry, Huashan Hospital, Fudan University, Shanghai, P.R. China

^2^Department of Infectious Diseases, Huashan Hospital, Fudan University, Shanghai, P.R. China

^3^Department of Dentistry, Zhongshan Hospital, Fudan University, Shanghai, P.R. China

*Correspondence: Liying Yu

Department of Dentistry

Huashan Hospital

Fudan University

Shanghai, 200040, P.R. China

Email: wuyu1984@hotmail.com

Jiazhen Chen

Department of Infectious Diseases

Huashan Hospital

Fudan University

Shanghai, 200040, P.R. China

Email: jiazhen_chen@163.com

**Appendix Figure 1.**

**
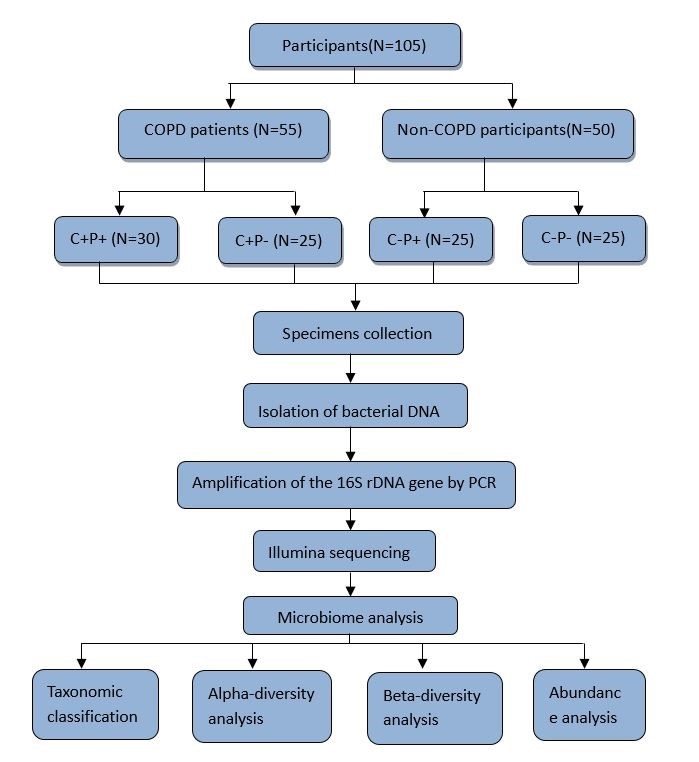
**

**Appendix Figure 1. Study design flowchart.** A total of 105 participants were recruited to the study, including 55 COPD patients and 50 non-COPD participants. After periodontal examination, 55 COPD patients were divided into two groups: 30 periodontitis patients with COPD (C+P+, N=30), and 25 COPD patients with no periodontitis (C+P-, N=25). Fifty non-COPD participants were divided into two groups: 25 periodontitis patients without COPD (C-P+, N=25), and 25 participants without COPD or periodontitis (C-P-, N=25). After periodontal samples were collected, bacterial DNA was isolated and sequenced. 16S rDNA sequencing data was analyzed for taxonomic classification, alpha-diversity analysis, beta-diversity analysis and abundance analysis.

**Appendix Figure 2.**


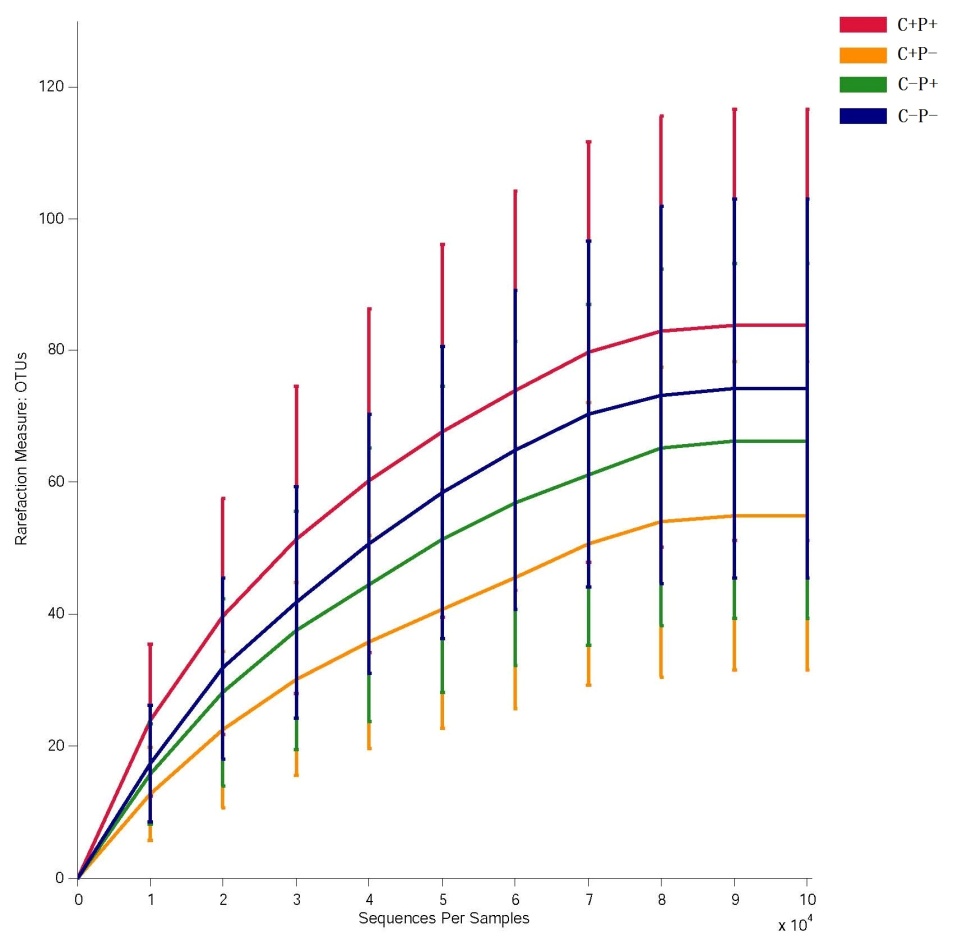


**Appendix Figure 2. Alpha diversity analysis based on subsampling analysis from four study groups.** Red lines indicate the C+P+ group, blue lines indicate the C+P- group, yellow lines indicate the C-P+ group and green lines indicate the C-P- group. Representative rarefaction curves depict richness (y-axis, as the number of OTUs), determined at an equal sampling effort (x-axis, the number of reads recovered in samples), of periodontal microbes. OTU reads of samples from each group were subsampled.

**Appendix Figure 3.**

**
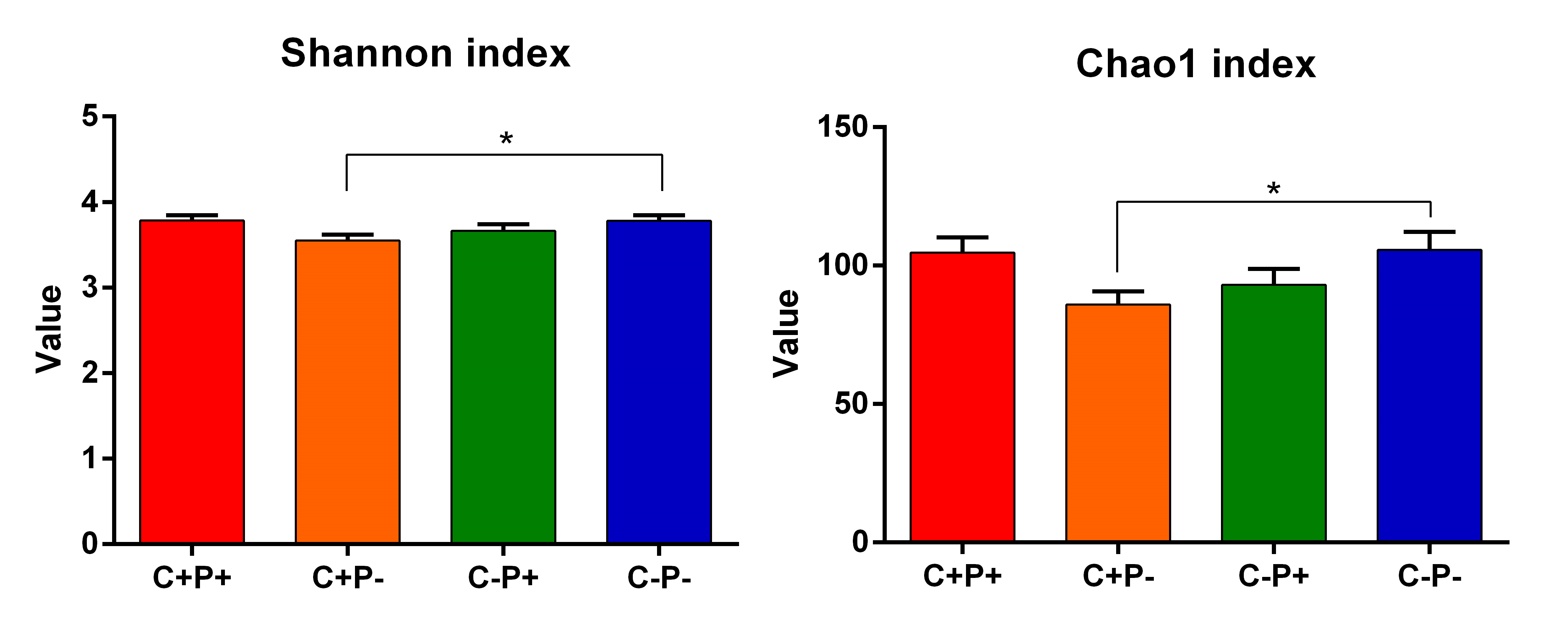
**

**Appendix Figure 3. The Shannon and Chao1 indexes of the four study groups.** Red bars indicate the C+P+ group, yellow bars indicate the C+P- group, green bars indicate the C-P+ group and blue bars indicate the C-P- group. A Mann-Whitney test was used to analyze the index between each two groups. Statistical significance in differences is indicated by the following: *: P<0.05, **: P<0.01, ***: P<0.001.

**Appendix Table 1. The most abundant bacterial genera detected and classified in 105 participants.**

| Genus | Biology | Reads (%) |
| --- | --- | --- |
| *Prevotella* | Gram-negative obligate anaerobes, dominant bacteria in gingival sulcus, suspicious pathogens of periodontitis. | 15.50 |
| *Treponema* | Gram-negative or difficult colored anaerobes or micro-aerobes. Can be isolated from the oral cavity, intestines and vagina. Oral resident bacteria, related to periodontitis and peri-implantitis. | 10.20 |
| *Streptococcus* | Gram-positive coccus widely distributed in nature. Many species are not pathogenic, and form part of the commensal human microbiota of the oral cavity, skin, intestine, and upper respiratory tract. | 8.85 |
| *Fusobacterium* | Gram-negative obligate anaerobic bacillus. Some are opportunistic pathogens that are related to periodontitis and topical skin ulcers. | 8.36 |
| *Porphyromonas* | Gram-negative obligate anaerobes. Pathogens in periodontitis and pulpitis, and related to rheumatoid arthritis. *P.gingivalis* is a “keystone” bacterium in the onset of chronic adult periodontitis. | 7.98 |
| *Neisseria* | Gram-negative aerobes that colonize the mucosal surfaces of many animals’ oral and nasopharyngeal cavity. Many species are not pathogenic except for *N.meningitidis* and *N.gonorrhoeae*. | 4.54 |
| *Leptotrichia* | Gram-negative anaerobic bacillus and a constituent of normal oral flora. | 2.85 |
| *Capnocytophaga* | Gram-negative facultative anaerobic bacillus that colonizes the oropharyngeal tract. A commensal species considered as an opportunistic pathogen. | 2.57 |
| *Selenomonas* | Gram-negative obligate anaerobic bacillus that can be isolated from gingival sulcus and periodontal pocket and colonize the gastrointestinal tracts. | 2.13 |
| *Veillonella* | Gram-negative anaerobes that are well known for lactate fermenting abilities. A normal bacterium in the intestine, upper respiratory tract and oral mucosa of mammals. | 1.98 |
| *Tannerella* | Gram-negative obligate anaerobes and pathogens to periodontitis. Related to atherosclerotic lesions and bacterial vaginosis. | 1.64 |
| *Parascardovia* | Gram-positive, with genus *Actinomyces* belong to one family. Can be isolated from dental caries and plaque. | 1.59 |
| *Filifactor* | With genus *Peptostreptococcus* belonging to one family, *F.alocis* is an important oral microbiota that is associated with periodontal disease and endodontic lesions. | 1.41 |
| *Mogibacterium* | Gram-positive obligate anaerobes isolated from the periodontal pockets with periodontal disease and infected root canals. | 1.38 |
| *Corynebacterium* | Gram-positive aerobes widely distributed in nature and mostly innocuous; one species, *C.diphtheriae* causes diphtheria. | 1.24 |
| *Gemella* | Gram-positive facultative anaerobes thrive best at high partial pressure of CO_2_, found in the mucous membranes of the oral cavity and upper digestive tract, with some related to pulmonary disease. | 1.21 |
| *Bacteroides* | Gram-negative obligate anaerobes constituting the most substantial portion of the gastrointestinal flora. Can be isolated in oral cavity and intestines. | 1.19 |
| *Actinomyces* | Gram-positive facultative anaerobes that are part of the healthy oral and vaginal microbiota. Dominant bacteria in supragingival plaque. | 0.82 |
| *Rothia* | Gram-positive facultative anaerobes that can be isolated from oral cavity. | 0.81 |
| *Lautropia* | Can be isolated from oral and nasal mucous. Decreased oral *Lautropia* is observed in esophageal squamous cell carcinoma patients. | 0.80 |
| *Candidatus Tammella* | Can be found in the gut of the termite. | 0.70 |
| *Dialister* | Belong to one family with genera *Veillonella* and *Selenomonas..* Can be isolated from oral cavity and intestines. | 0.51 |

Data were obtained from the NCBI database and one of its LinkOut Providers (Encyclopedia of Life). Abundance cutoff in this table was set at 0.5%. Some abundance cutoffs were below 0.5% and are shown in Appendix Table 2.

**Appendix Table 2**. **The remaining 47 bacterial genera detected and classified in 105 participants.**

| Genus | Reads (%) | Genus | Reads (%) |
| --- | --- | --- | --- |
| *Johnsonella* | 0.47 | *Acetobacterium* | 0.19 |
| *Sedimentibacter* | 0.45 | *Elizabethkingia* | 0.19 |
| *Desulfobulbus* | 0.45 | *Chryseobacterium* | 0.19 |
| *Thermovirga* | 0.45 | *Longilinea* | 0.18 |
| *Peptostreptococcus* | 0.44 | *Eikenella* | 0.17 |
| *Catonella* | 0.37 | *Desulfomicrobium* | 0.17 |
| *Odoribacter* | 0.35 | *Dysgonomonas* | 0.17 |
| *Megasphaera* | 0.32 | *Bacillus* | 0.17 |
| *Campylobacter* | 0.31 | *Peptococcus* | 0.16 |
| *Granulicatella* | 0.31 | *Aggregatibacter* | 0.16 |
| *Clostridium* | 0.30 | *Moryella* | 0.14 |
| *Kingella* | 0.29 | *Haemophilus* | 0.14 |
| *Hylemonella* | 0.28 | *Arcanobacterium* | 0.14 |
| *Butyrivibrio* | 0.27 | *Brevundimonas* | 0.14 |
| *Sphingobacterium* | 0.27 | *Pseudoramibacter* | 0.13 |
| *Sneathia* | 0.26 | *Delftia* | 0.13 |
| *Mycoplasma* | 0.26 | *Propionibacterium* | 0.13 |
| *Pectinatus* | 0.26 | *Oribacterium* | 0.13 |
| *Mitsuokella* | 0.26 | *Luteococcus* | 0.13 |
| *Alkaliphilus* | 0.25 | *Sebaldella* | 0.13 |
| *Candidatus Blochmannia* | 0.24 | *Blautia* | 0.12 |
| *Paenibacillus* | 0.23 | *Flavobacterium* | 0.12 |
| *Pyrobaculum* | 0.22 | *Actinobaculum* | 0.11 |
| *Atopobium* | 0.20 |  |  |

**Appendix Table 2B. The bacterial species detected and classified in 105 participants.**

| Species | Reads (%) | Species | Reads (%) |
| --- | --- | --- | --- |
| *Porphyromonas gingivalis* | 4.78 | *Megasphaera geminatus* | 0.29 |
| *Fusobacterium nucleatum* | 4.63 | *Treponema lecithinolyticum* | 0.28 |
| *Prevotella intermedia* | 3.74 | *Leptotrichia wadei* | 0.28 |
| *Treponema medium* | 3.51 | *Odoribacter denticanis* | 0.27 |
| *Prevotella tannerae* | 2.74 | *Johnsonella ignava* | 0.26 |
| *Treponema denticola* | 2.46 | *Sphingobacterium shayense* | 0.26 |
| *Porphyromonas endodontalis* | 2.26 | *Sneathia sanguinegens* | 0.26 |
| *Fusobacterium naviforme* | 1.64 | *Pectinatus cerevisiiphilus* | 0.26 |
| *Streptococcus tigurinus* | 1.53 | *Alkaliphilus crotonatoxidans* | 0.25 |
| *Tannerella forsythia* | 1.45 | *Prevotella dentalis* | 0.24 |
| *Prevotella loescheii* | 1.42 | *Candidatus Blochmannia rufipes* | 0.24 |
| *Filifactor alocis* | 1.40 | *Bacteroides heparinolyticus* | 0.23 |
| *Streptococcus pseudopneumoniae* | 1.40 | *Kingella oralis* | 0.23 |
| *Neisseria lactamica* | 1.33 | *Streptococcus milleri* | 0.23 |
| *Prevotella oris* | 1.13 | *Pyrobaculum neutrophilum* | 0.22 |
| *Neisseria mucosa* | 1.11 | *Granulicatella adiacens* | 0.22 |
| *Corynebacterium matruchotii* | 1.05 | *Streptococcus anginosus* | 0.21 |
| *Veillonella atypica* | 1.05 | *Mogibacterium timidum* | 0.20 |
| *Treponema socranskii* | 0.98 | *Campylobacter showae* | 0.19 |
| *Prevotella nigrescens* | 0.84 | *Rothia dentocariosa* | 0.19 |
| *Treponema maltophilum* | 0.78 | *Gemella sanguinis* | 0.18 |
| *Neisseria flavescens* | 0.74 | *Elizabethkingia meningoseptica* | 0.18 |
| *Treponema amylovorum* | 0.73 | *Veillonella parvula* | 0.18 |
| *Neisseria elongata* | 0.71 | *Atopobium rimae* | 0.18 |
| *Streptococcus intermedius* | 0.70 | *Longilinea arvoryzae* | 0.18 |
| *Selenomonas infelix* | 0.65 | *Porphyromonas canis* | 0.18 |
| *Candidatus Tammella caduceiae* | 0.64 | *Actinomyces georgiae* | 0.17 |
| *Capnocytophaga ochracea* | 0.62 | *Dysgonomonas wimpennyi* | 0.17 |
| *Streptococcus oralis* | 0.61 | *Desulfomicrobium orale* | 0.17 |
| *Gemella haemolysans* | 0.58 | *Prevotella maculosa* | 0.16 |
| *Lautropia mirabilis* | 0.57 | *Actinomyces cardiffensis* | 0.16 |
| *Streptococcus infantis* | 0.51 | *Treponema vincentii* | 0.16 |
| *Dialister invisus* | 0.51 | *Prevotella denticola* | 0.16 |
| *Prevotella pleuritidis* | 0.45 | *Fusobacterium simiae* | 0.16 |
| *Sedimentibacter hydroxybenzoicus* | 0.45 | *Actinomyces odontolyticus* | 0.16 |
| *Thermovirga lienii* | 0.45 | *Treponema putidum* | 0.16 |
| *Prevotella multiformis* | 0.45 | *Rothia aeria* | 0.15 |
| *Bacteroides graminisolvens* | 0.44 | *Mycoplasma faucium* | 0.15 |
| *Veillonella dispar* | 0.44 | *Actinomyces meyeri* | 0.14 |
| *Peptostreptococcus stomatis* | 0.44 | *Capnocytophaga leadbetteri* | 0.13 |
| *Treponema succinifaciens* | 0.42 | *Streptococcus bovis* | 0.13 |
| *Capnocytophaga granulosa* | 0.39 | *Bacteroides denticanum* | 0.13 |
| *Selenomonas noxia* | 0.37 | *Brevundimonas staleyi* | 0.13 |
| *Streptococcus gordonii* | 0.36 | *Catonella morbi* | 0.13 |
| *Prevotella melaninogenica* | 0.35 | *Luteococcus peritonei* | 0.13 |
| *Streptococcus sanguinis* | 0.34 | *Butyrivibrio proteoclasticus* | 0.13 |
| *Porphyromonas catoniae* | 0.34 | *Streptococcus australis* | 0.13 |
| *Streptococcus vestibularis* | 0.34 | *Corynebacterium durum* | 0.12 |
| *Leptotrichia buccalis* | 0.33 | *Prevotella baroniae* | 0.12 |
| *Prevotella oulorum* | 0.33 | *Prevotella buccae* | 0.11 |
| *Streptococcus cristatus* | 0.33 | *Leptotrichia hofstadii* | 0.11 |
| *Selenomonas artemidis* | 0.33 | *Streptococcus oligofermentans* | 0.11 |
| *Capnocytophaga gingivalis* | 0.32 | *Propionibacterium humerusii* | 0.11 |
| *Prevotella veroralis* | 0.31 | *Treponema paraluiscuniculi* | 0.10 |
| *Leptotrichia shahii* | 0.30 | *Gemella cunicula* | 0.10 |
| *Prevotella paludivivens* | 0.30 |  |  |

Abundance cutoff was set at 0.1%. Values below 0.1% were not shown in the table.

**Appendix Table 3. Comparison of bacterial genera and species between COPD and non-COPD patients.**

| Genus and Species | P-value | COPD (mean±se) | | | non-COPD (mean±se) | | |
| --- | --- | --- | --- | --- | --- | --- | --- |
| *Johnsonella* | * | 0.58 | ± | 0.10 | 0.27 | ± | 0.06 |
| *Catonella* | * | 0.51 | ± | 0.09 | 0.28 | ± | 0.05 |
| *Campylobacter* | * | 0.38 | ± | 0.06 | 0.22 | ± | 0.05 |
| *Oribacterium* | * | 0.08 | ± | 0.02 | 0.19 | ± | 0.05 |
| *Arcanobacterium* | ** | 0.07 | ± | 0.02 | 0.22 | ± | 0.05 |
| *Streptomyces* | * | 0.05 | ± | 0.01 | 0.12 | ± | 0.02 |
| *Prevotella intermedia* | * | 4.57 | ± | 0.84 | 2.34 | ± | 0.54 |
| *Prevotella tannerae* | * | 1.64 | ± | 0.43 | 3.54 | ± | 0.72 |
| *Johnsonella ignava* | * | 0.32 | ± | 0.05 | 0.17 | ± | 0.03 |
| *Porphyromonas canis* | ** | 0.27 | ± | 0.04 | 0.11 | ± | 0.03 |
| *Fusobacterium simiae* | * | 0.26 | ± | 0.06 | 0.08 | ± | 0.02 |
| *Campylobacter showae* | * | 0.25 | ± | 0.05 | 0.12 | ± | 0.03 |
| *Gemella sanguinis* | * | 0.24 | ± | 0.07 | 0.08 | ± | 0.02 |
| *Prevotella melaninogenica* | * | 0.23 | ± | 0.05 | 0.46 | ± | 0.09 |
| *Veillonella dispar* | * | 0.20 | ± | 0.06 | 0.77 | ± | 0.23 |
| *Streptococcus gordonii* | * | 0.19 | ± | 0.05 | 0.65 | ± | 0.19 |
| *Prevotella oulorum* | * | 0.18 | ± | 0.08 | 0.52 | ± | 0.14 |
| *Catonella morbi* | * | 0.17 | ± | 0.03 | 0.08 | ± | 0.02 |
| *Gemella cunicula* | * | 0.13 | ± | 0.03 | 0.05 | ± | 0.01 |
| *Arcanobacterium bernardiae* | ** | 0.04 | ± | 0.01 | 0.13 | ± | 0.03 |
| *Streptococcus parasanguinis* | * | 0.01 | ± | 0.01 | 0.15 | ± | 0.07 |

Abundance cutoff was set at 0.1%. Values below 0.1% were not shown in the table.

*: P<0.05, **: P<0.01, ***: P<0.001.

**Appendix Table 4. Average relative abundance of forty-three bacterial genera with significant statistical differences between C-P+ and C-P-patients.**

| Genus | P-value | (C-P+) ± se | | | (C-P-) ± se | | |
| --- | --- | --- | --- | --- | --- | --- | --- |
| *Treponema* | *** | 14.62 | ± | 1.60 | 4.77 | ± | 1.48 |
| *Porphyromonas* | *** | 11.59 | ± | 1.72 | 3.67 | ± | 1.04 |
| *Fusobacterium* | * | 9.13 | ± | 0.95 | 6.30 | ± | 0.93 |
| *Streptococcus* | *** | 3.32 | ± | 0.79 | 15.77 | ± | 2.55 |
| *Tannerella* | *** | 3.25 | ± | 0.65 | 0.66 | ± | 0.16 |
| *Filifactor* | *** | 2.25 | ± | 0.32 | 0.43 | ± | 0.18 |
| *Mogibacterium* | *** | 2.22 | ± | 0.40 | 0.59 | ± | 0.17 |
| *Candidatus Tammella* | *** | 1.30 | ± | 0.23 | 0.17 | ± | 0.07 |
| *Veillonella* | ** | 1.29 | ± | 0.56 | 4.42 | ± | 1.25 |
| *Desulfobulbus* | *** | 1.15 | ± | 0.28 | 0.06 | ± | 0.04 |
| *Neisseria* | *** | 1.03 | ± | 0.41 | 6.58 | ± | 1.40 |
| *Capnocytophaga* | ** | 1.03 | ± | 0.35 | 4.56 | ± | 1.27 |
| *Dialister* | * | 0.88 | ± | 0.25 | 0.30 | ± | 0.10 |
| *Thermovirga* | *** | 0.78 | ± | 0.12 | 0.13 | ± | 0.06 |
| *Sedimentibacter* | *** | 0.76 | ± | 0.17 | 0.10 | ± | 0.05 |
| *Parascardovia* | *** | 0.74 | ± | 0.19 | 2.80 | ± | 0.42 |
| *Odoribacter* | ** | 0.70 | ± | 0.26 | 0.02 | ± | 0.02 |
| *Corynebacterium* | ** | 0.57 | ± | 0.25 | 2.52 | ± | 0.85 |
| *Longilinea* | *** | 0.49 | ± | 0.13 | 0.04 | ± | 0.03 |
| *Actinomyces* | *** | 0.44 | ± | 0.10 | 1.49 | ± | 0.26 |
| *Catonella* | * | 0.37 | ± | 0.07 | 0.19 | ± | 0.06 |
| *Kingella* | ** | 0.37 | ± | 0.36 | 0.22 | ± | 0.09 |
| *Acetobacterium* | ** | 0.36 | ± | 0.09 | 0.05 | ± | 0.02 |
| *Pectinatus* | *** | 0.34 | ± | 0.07 | 0.09 | ± | 0.03 |
| *Pseudoramibacter* | * | 0.34 | ± | 0.24 | 0.03 | ± | 0.02 |
| *Lautropia* | *** | 0.25 | ± | 0.17 | 1.96 | ± | 0.54 |
| *Peptoniphilus* | ** | 0.23 | ± | 0.09 | 0.01 | ± | 0.01 |
| *Luteococcus* | * | 0.19 | ± | 0.18 | 0.16 | ± | 0.08 |
| *Aminobacterium* | *** | 0.19 | ± | 0.05 | 0.01 | ± | 0.01 |
| *Gemella* | *** | 0.19 | ± | 0.07 | 1.07 | ± | 0.28 |
| *Slackia* | * | 0.12 | ± | 0.03 | 0.03 | ± | 0.01 |
| *Granulicatella* | ** | 0.12 | ± | 0.05 | 0.67 | ± | 0.17 |
| *Dysgonomonas* | * | 0.11 | ± | 0.04 | 0.01 | ± | 0.00 |
| *Rothia* | *** | 0.10 | ± | 0.06 | 1.93 | ± | 0.98 |
| *Arcanobacterium* | *** | 0.07 | ± | 0.05 | 0.37 | ± | 0.08 |
| *Actinobaculum* | * | 0.06 | ± | 0.02 | 0.22 | ± | 0.08 |
| *Streptomyces* | ** | 0.06 | ± | 0.03 | 0.18 | ± | 0.04 |
| *Chryseobacterium* | ** | 0.04 | ± | 0.03 | 0.22 | ± | 0.08 |
| *Haemophilus* | ** | 0.01 | ± | 0.01 | 0.24 | ± | 0.10 |
| *Propionibacterium* | ** | 0.00 | ± | 0.00 | 0.31 | ± | 0.18 |
| *Blastomonas* | * | 0.00 | ± | 0.00 | 0.13 | ± | 0.11 |
| *Cardiobacterium* | * | 0.00 | ± | 0.00 | 0.12 | ± | 0.10 |
| *Elizabethkingia* | * | 0.00 | ± | 0.00 | 0.11 | ± | 0.08 |

Abundance cutoff was set at 0.1%. Values below 0.1% were not shown in the table.

*: P<0.05, **: P<0.01, ***: P<0.001.

**Appendix Table 5. Average relative abundance of seventy-nine bacterial species with significant statistical differences between C-P+ and C-P-patients.**

| Species | P-value | (C-P+) ± se | | | (C-P-) ± se | | |
| --- | --- | --- | --- | --- | --- | --- | --- |
| *Porphyromonas gingivalis* | *** | 8.87 | ± | 1.74 | 1.32 | ± | 0.68 |
| *Fusobacterium nucleatum* | * | 4.76 | ± | 0.52 | 3.39 | ± | 0.54 |
| *Treponema denticola* | *** | 4.28 | ± | 0.56 | 0.80 | ± | 0.26 |
| *Treponema medium* | ** | 4.11 | ± | 0.59 | 1.82 | ± | 0.58 |
| *Prevotella intermedia* | * | 3.48 | ± | 0.89 | 1.21 | ± | 0.54 |
| *Tannerella forsythia* | *** | 3.12 | ± | 0.66 | 0.40 | ± | 0.15 |
| *Filifactor alocis* | *** | 2.24 | ± | 0.32 | 0.43 | ± | 0.18 |
| *Porphyromonas endodontalis* | ** | 2.19 | ± | 0.36 | 1.27 | ± | 0.49 |
| *Treponema socranskii* | ** | 1.42 | ± | 0.20 | 0.61 | ± | 0.16 |
| *Treponema maltophilum* | *** | 1.33 | ± | 0.21 | 0.31 | ± | 0.10 |
| *Candidatus Tammella caduceiae* | *** | 1.15 | ± | 0.18 | 0.17 | ± | 0.07 |
| *Dialister invisus* | * | 0.88 | ± | 0.25 | 0.30 | ± | 0.10 |
| *Treponema amylovorum* | * | 0.83 | ± | 0.19 | 0.43 | ± | 0.18 |
| *Thermovirga lienii* | *** | 0.78 | ± | 0.12 | 0.13 | ± | 0.06 |
| *Sedimentibacter hydroxybenzoicus* | *** | 0.76 | ± | 0.17 | 0.10 | ± | 0.05 |
| *Veillonella atypica* | *** | 0.66 | ± | 0.32 | 2.13 | ± | 0.62 |
| *Treponema succinifaciens* | * | 0.58 | ± | 0.24 | 0.27 | ± | 0.20 |
| *Odoribacter denticanis* | ** | 0.52 | ± | 0.19 | 0.02 | ± | 0.02 |
| *Longilinea arvoryzae* | *** | 0.49 | ± | 0.13 | 0.04 | ± | 0.03 |
| *Corynebacterium matruchotii* | * | 0.48 | ± | 0.20 | 2.10 | ± | 0.81 |
| *Streptococcus tigurinus* | *** | 0.48 | ± | 0.17 | 2.05 | ± | 0.34 |
| *Streptococcus pseudopneumoniae* | *** | 0.36 | ± | 0.08 | 2.35 | ± | 0.46 |
| *Pectinatus cerevisiiphilus* | *** | 0.34 | ± | 0.07 | 0.09 | ± | 0.03 |
| *Kingella oralis* | * | 0.34 | ± | 0.33 | 0.10 | ± | 0.03 |
| *Prevotella dentalis* | * | 0.30 | ± | 0.13 | 0.09 | ± | 0.07 |
| *Treponema putidum* | *** | 0.30 | ± | 0.08 | 0.02 | ± | 0.01 |
| *Capnocytophaga ochracea* | ** | 0.27 | ± | 0.13 | 1.40 | ± | 0.50 |
| *Neisseria lactamica* | *** | 0.26 | ± | 0.12 | 2.24 | ± | 0.58 |
| *Capnocytophaga granulosa* | * | 0.25 | ± | 0.12 | 0.68 | ± | 0.29 |
| *Veillonella dispar* | ** | 0.25 | ± | 0.12 | 1.29 | ± | 0.42 |
| *Mogibacterium timidum* | ** | 0.25 | ± | 0.05 | 0.07 | ± | 0.03 |
| *Streptococcus cristatus* | * | 0.25 | ± | 0.12 | 0.64 | ± | 0.21 |
| *Bacteroides heparinolyticus* | * | 0.23 | ± | 0.09 | 0.01 | ± | 0.01 |
| *Peptoniphilus indolicus* | ** | 0.23 | ± | 0.09 | 0.01 | ± | 0.01 |
| *Neisseria mucosa* | ** | 0.22 | ± | 0.11 | 1.40 | ± | 0.55 |
| *Atopobium rimae* | * | 0.22 | ± | 0.09 | 0.21 | ± | 0.15 |
| *Treponema vincentii* | * | 0.21 | ± | 0.07 | 0.14 | ± | 0.10 |
| *Leptotrichia shahii* | * | 0.20 | ± | 0.07 | 0.38 | ± | 0.08 |
| *Luteococcus peritonei* | * | 0.19 | ± | 0.18 | 0.16 | ± | 0.08 |
| *Aminobacterium colombiense* | *** | 0.19 | ± | 0.05 | 0.01 | ± | 0.01 |
| *Lautropia mirabilis* | *** | 0.18 | ± | 0.14 | 1.29 | ± | 0.36 |
| *Treponema paraluiscuniculi* | ** | 0.16 | ± | 0.03 | 0.03 | ± | 0.01 |
| *Porphyromonas catoniae* | * | 0.16 | ± | 0.08 | 0.35 | ± | 0.13 |
| *Bacteroides denticanum* | ** | 0.15 | ± | 0.03 | 0.05 | ± | 0.03 |
| *Treponema porcinum* | * | 0.14 | ± | 0.03 | 0.04 | ± | 0.02 |
| *Streptococcus oralis* | *** | 0.12 | ± | 0.04 | 0.68 | ± | 0.13 |
| *Catonella morbi* | * | 0.12 | ± | 0.03 | 0.05 | ± | 0.02 |
| *Gemella haemolysans* | *** | 0.11 | ± | 0.04 | 0.54 | ± | 0.17 |
| *Dysgonomonas wimpennyi* | * | 0.11 | ± | 0.04 | 0.01 | ± | 0.00 |
| *Neisseria flavescens* | * | 0.10 | ± | 0.04 | 0.93 | ± | 0.38 |
| *Streptococcus oligofermentans* | * | 0.08 | ± | 0.06 | 0.18 | ± | 0.06 |
| *Streptococcus infantis* | *** | 0.08 | ± | 0.02 | 0.73 | ± | 0.14 |
| *Veillonella parvula* | ** | 0.08 | ± | 0.04 | 0.51 | ± | 0.21 |
| *Streptococcusgordonii* | *** | 0.07 | ± | 0.04 | 1.23 | ± | 0.34 |
| *Granulicatella adiacens* | *** | 0.07 | ± | 0.04 | 0.42 | ± | 0.10 |
| *Actinomyces georgiae* | ** | 0.07 | ± | 0.03 | 0.24 | ± | 0.05 |
| *Actinomyces odontolyticus* | * | 0.07 | ± | 0.03 | 0.30 | ± | 0.09 |
| *Actinomyces meyeri* | ** | 0.05 | ± | 0.03 | 0.21 | ± | 0.05 |
| *Arcanobacterium bernardiae* | ** | 0.05 | ± | 0.02 | 0.22 | ± | 0.05 |
| *Corynebacterium durum* | ** | 0.04 | ± | 0.03 | 0.28 | ± | 0.10 |
| *Streptococcus australis* | ** | 0.04 | ± | 0.02 | 0.44 | ± | 0.17 |
| *Capnocytophaga gingivalis* | ** | 0.03 | ± | 0.02 | 1.15 | ± | 0.50 |
| *Actinobaculum suis* | ** | 0.03 | ± | 0.01 | 0.13 | ± | 0.04 |
| *Streptococcus bovis* | *** | 0.03 | ± | 0.02 | 0.30 | ± | 0.08 |
| *Leptotrichia buccalis* | ** | 0.02 | ± | 0.02 | 0.39 | ± | 0.16 |
| *Streptococcus sanguinis* | *** | 0.02 | ± | 0.02 | 0.55 | ± | 0.15 |
| *Streptococcus vestibularis* | *** | 0.02 | ± | 0.01 | 1.47 | ± | 0.71 |
| *Bacteroides graminisolvens* | ** | 0.02 | ± | 0.02 | 1.10 | ± | 0.69 |
| *Rothia aeria* | *** | 0.02 | ± | 0.02 | 0.22 | ± | 0.06 |
| *Gemella sanguinis* | ** | 0.02 | ± | 0.01 | 0.15 | ± | 0.04 |
| *Prevotella saccharolytica* | * | 0.02 | ± | 0.01 | 0.20 | ± | 0.07 |
| *Leptotrichia hofstadii* | * | 0.02 | ± | 0.01 | 0.12 | ± | 0.04 |
| *Streptococcus parasanguinis* | * | 0.01 | ± | 0.01 | 0.29 | ± | 0.13 |
| *Haemophilus parainfluenzae* | ** | 0.01 | ± | 0.01 | 0.23 | ± | 0.09 |
| *Streptococcus fryi* | ** | 0.00 | ± | 0.00 | 0.13 | ± | 0.05 |
| *Campylobacter gracilis* | ** | 0.00 | ± | 0.00 | 0.11 | ± | 0.04 |
| *Propionibacterium humerusii* | ** | 0.00 | ± | 0.00 | 0.24 | ± | 0.17 |
| *Blastomonas natatoria* | * | 0.00 | ± | 0.00 | 0.13 | ± | 0.11 |
| *Elizabethkingia meningoseptica* | * | 0.00 | ± | 0.00 | 0.11 | ± | 0.08 |

Abundance cutoff was set at 0.1%. Values below 0.1% were not shown in the table.

*: P<0.05, **: P<0.01, ***: P<0.001.
